# Astragalus Membranaceus—Can It Delay Cellular Aging?

**DOI:** 10.3390/nu17081299

**Published:** 2025-04-08

**Authors:** Kinga K. Borowicz, Monika E. Jach

**Affiliations:** 1 Independent Experimental Neuropathophysiology Unit, Department of Toxicology, Faculty of Pharmacy, Medical University of Lublin, Jaczewskiego 8b Street, 20-090 Lublin, Poland; 2 Department of Molecular Biology, The John Paul II Catholic University of Lublin, Konstantynów Street 1I, 20-708 Lublin, Poland; monika.jach@kul.pl

**Keywords:** *Astragalus membranaceus*, anti-aging effects, cellular senescence, skin aging, systemic aging

## Abstract

*Astragalus membranaceus*, a plant that has been utilized in traditional Chinese medicine for centuries, is widely regarded as one of the most valuable herbs in this medicinal tradition. It is commonly referred to as the “yellow leader”, a designation that stems from the yellow hue of its most significant organ, the root, and its adaptogenic properties. The plant Astragalus is renowned for its abundance of active components, including polysaccharides, flavonoids, saponins, and an array of trace elements. It has been demonstrated that the administration of Astragalus can prevent cellular aging, owing to its diverse range of actions that provide protection to the body from both external and internal factors. The antioxidant, immunomodulatory, anti-inflammatory, and regenerative properties of this plant contribute to the maintenance of good skin condition, preventing atrophy of subcutaneous tissue and degeneration of facial bones. Systemic actions encompass the maintenance of function and protection of the cardiovascular, nervous, respiratory, digestive, excretory, immune, and endocrine systems. This article reviews the composition of *Astragalus membranaceus* and the beneficial effects of its root extract and its active substances on the whole body, with a particular focus on the anti-aging effects on the skin.

## 1. Introduction

**Figure 1:**
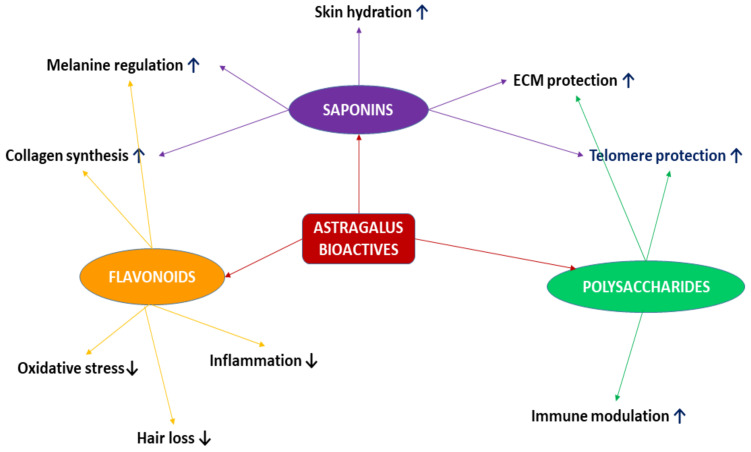
The anti-aging effects of the active ingredients of Astragalus on the skin and skin appendages.

The herb *Astragalus membranaceus* (syn. *Astragalus mongholicus*, Mongolian milkvetch, Huangqi) is regarded as one of the most valuable and ancient medicinal plants in traditional Asian medicine. The root of Astragalus, considered the primary organ of importance, contains a plethora of valuable active ingredients, numbering over 200. These include polysaccharides, saponins, and flavonoids. The most widely recognized property of Astragalus extract is its adaptogenic and tonic effect, which provides support to the body’s vital forces. The systemic effects of this extract include expectorant, diuretic, immunomodulatory, antimicrobial, and anticancer properties. Its ability to enhance the optimal functioning of the liver, kidneys, heart muscle, blood vessels, and brain has been demonstrated. Notably, the most significant interest in the Western world has been in the anti-aging effects of Astragalus both in the skin (Figure 1) and systemic organs. This is particularly salient in populations experiencing an accelerating rate of aging, which is accompanied by an increase in the prevalence of chronic diseases, a reduction in the body’s capacity to adapt to changing environmental factors, and a decline in functional capacity. Consequently, there is a growing interest in agents that retard cellular aging, with the expectation that this will result in a deceleration of aging and degenerative processes throughout the body. The concept of anti-aging is regarded as a means to augment the period during which individuals can maintain optimal health and well-being (health span) [1].

This review focuses on the anti-aging effects of Astragalus on the skin. The skin, being an external organ, serves as a reflection of the internal aging processes. As individuals undergo the aging process, the skin undergoes changes, including the loss of firmness and elasticity, stretching, the formation of wrinkles, and dryness. Submission to the law of gravity leads to the formation of wrinkles, disrupting the facial proportions and oval shape. Clinical research has demonstrated that *Astragalus membranaceus* exerts a beneficial effect on skin condition, attributable to its antioxidant, anti-inflammatory, and telomere protection properties (Figure 1). It is hypothesized that the benefits of Astragalus can be extended to other tissues, organs, and systems, potentially extending their lifespan. However, to date, there is no evidence that Astragalus can extend the lifespan of the entire organism [2].

## 2. Active Ingredients and Their Brief Characteristics

A total of 100-plus chemically active compounds have been identified in *Astragalus membranaceus*, including flavonoids, saponins, and polysaccharides [3,4].

### 2.1. Flavonoids

A plethora of active compounds have been identified in Astragalus, with approximately 30 flavonoids belonging to the structural groups of flavones, isoflavones, isoflavanones, and pterocarpanes having been isolated. The most significant biologically active flavonoids include calycosin, calycosin-7-O-β-D-glucoside, formononetin, ononin, (3R)-8,2′-dihydroxy-7, 4′-dimethoxyisoflavan, 9,10-dimethoxypterocarpan-3-O-β-D-glucoside, 3-hydroxy-9,10-dimethoxypterocarpan, quercetin, and kaempherol [5].

Calycosin has demonstrated antihypertensive and neuroprotective effects and proved its effectiveness in the prevention and treatment of cardiovascular and neurodegenerative diseases. Calycosin-7-O-β-D-glucopyranoside has proven antiviral activity and is employed in the treatment of viral myocarditis [6]. Its capacity to impede cell apoptosis and the inflammatory response triggered by advanced glycation products enables it to enhance the condition of endothelial cells. Formononetin has a positive impact on the skeletal system, protecting osteoblasts and stimulating their osteogenic differentiation. Additionally, formononetin exhibits antioxidant, phytoestrogenic, and anti-inflammatory effects. Quercetin has been demonstrated to possess primarily neuroprotective, cardioprotective, and anti-atherosclerotic effects. Moreover, its antioxidant, anti-inflammatory, antiviral, and immunomodulatory properties have been well-established. The efficacy of kaempferol has been shown in the treatment of metabolic diseases, including atherosclerosis, diabetes mellitus, obesity, and non-alcoholic fatty liver disease [7,8].

### 2.2. Triterpenoid Saponins

A total of approximately 40 saponins have been extracted from the root of Astragalus. The majority of these compounds are classified as cycloartane tetracyclic triterpenoids. The most significant biologically active saponins include astragaloside I, astragaloside II, astragaloside IV, isoastragaloside I, isoastragaloside II, acetylastragaloside I, astramembrannin II, and soyasaponin I. Astragaloside IV is the primary active component of the root and serves as a marker for assessing the quality of raw plant material. Its benefits have been demonstrated to include positive effects on organ function, as well as anti-inflammatory, antiviral, and hypoglycemic effects. In addition, astragaloside IV has been shown to have a positive effect on the immune system and cell apoptosis [3,9]. The pharmacological action of astragaloside IV encompasses anti-inflammatory, antioxidant, neuroprotective, antifibrotic, mitochondrial gene mutation regulation, and antitumor effects [5].

### 2.3. Polysaccharides

The polysaccharides present in Astragalus have been demonstrated to exert a range of beneficial effects, including immunomodulatory, anti-aging, anti-inflammatory, antidiabetic, lipid-lowering, anti-tumor, antifibrotic, and antiviral properties. With regard to the antidiabetic effects of these substances, there is evidence that they can reduce insulin resistance. In the context of traditional Chinese medicine, polysaccharides have been utilized as an adjunctive treatment for various ailments, including acute myocarditis, nephritis, and type 2 diabetes mellitus [6,10,11].

### 2.4. Other Chemical Compounds

A review of the literature reveals that Astragalus contains approximately 20 microelements, including scandium, chromium, cobalt, copper, selenium, molybdenum, cesium, iron, manganese, zinc, and rubidium. In addition, as a member of the Fabaceae family, it is a notable source of amino acids. Of the 20 identified amino acids, asparagine, canavanine, proline, arginine, aspartic acid, and alanine are the most abundant. Additionally, the root of Astragalus contains substantial amounts of coumarin, folic acid, choline, betaine, linoleic acid, linolenic acid, vanillic acid, ferulic acid, isoferulic acid, palmitic acid, hydroxyphenylacrylic acid, caffeic acid, green acid, palmic acid, 13-sitosterol, daucosterol, lupeol, and numerous other compounds [3,9].

### 2.5. Selected Physicochemical Properties of Bioactive Compounds in Astragalus membranaceus

*Astragalus membranaceus* is widely known for its pharmacological properties, attributed to several active constituents, including astragaloside IV, cycloastragenol, calycosin, formononetin, quercetin, and polysaccharides such as β-(1→3)-D-glucan. The following is an overview of their physicochemical characteristics.

Astragaloside IV is a cycloartane-type triterpene glycoside with the molecular formula C_41_H_68_O_14_ and a molecular weight of 784.97 g/mol. It appears as a white powder with a melting point of 284–285 °C. Due to its glycosidic nature, it is sparingly soluble in water but readily soluble in methanol and ethanol. Astragaloside IV is hydrolyzed to its aglycone, cycloastragenol, which is considered a key bioactive metabolite. Cycloastragenol is a tetracyclic triterpenoid sapogenin, structurally related to astragaloside IV, with a molecular formula of C_30_H_50_O_5_ and a molecular weight of 490.72 g/mol. It is typically found as a white crystalline powder with a melting point of 240–241 °C.

Cycloastragenol is practically insoluble in water but dissolves in organic solvents such as chloroform, methanol, and ethanol. It has gained attention for its potential telomerase-activating properties, which may contribute to its anti-aging effects.

Calycosin is a naturally occurring isoflavone with the molecular formula C_16_H_12_O_5_ and a molecular weight of 284.26 g/mol. It presents as a yellow crystalline powder with a melting point of 186–188 °C. Calycosin is slightly soluble in water but dissolves readily in organic solvents such as ethanol and acetone. It is one of the major flavonoids in *Astragalus membranaceus*, known for its antioxidant and estrogenic activity.

Formononetin is an O-methylated isoflavone, structurally related to calycosin, with a molecular formula of C_16_H_12_O_4_ and a molecular weight of 268.26 g/mol. It appears as a white to off-white crystalline powder with a melting point of 256–258 °C. Like calycosin, it is insoluble in water but soluble in ethanol, methanol, and acetone. Formononetin is classified as a phytoestrogen, contributing to the estrogenic activity of *Astragalus membranaceus*.

Quercetin is a widely distributed flavonol with a molecular formula of C_15_H_10_O_7_ and a molecular weight of 302.24 g/mol. It is a yellow crystalline powder that decomposes at approximately 314 °C. Quercetin is poorly soluble in water, but it dissolves in ethanol, DMSO, and alkaline aqueous solutions. Due to its strong antioxidant and anti-inflammatory properties, it is frequently investigated for its role in oxidative stress regulation.

β-(1→3)-D-glucan is a high-molecular-weight polysaccharide and a key immunomodulatory component of *Astragalus membranaceus*. Its molecular formula is (C_6_H_10_O_5_)n, and its structure consists of glucose monomers linked primarily by β-(1→3) glycosidic bonds, with some β-(1→6) branching. APS are water-soluble, forming viscous solutions, and they exhibit significant biological stability. They are recognized for their immunostimulatory, anti-inflammatory, and antioxidant effects, acting through the modulation of macrophages and cytokine signaling pathways.

The bioactive constituents of *Astragalus membranaceus* exhibit diverse physicochemical properties that influence their solubility, bioavailability, and pharmacological activities. The presence of triterpene glycosides (astragaloside IV and cycloastragenol), flavonoids (calycosin, formononetin, and quercetin), and high-molecular-weight polysaccharides (β-(1→3)-D-glucan) contributes to the plant’s antioxidant, immunomodulatory, and potential anti-aging effects.

## 3. Aging of the Skin

The human skin is a reflection of the processes occurring within the body. The aging process is influenced by a number of agents. Among the external factors that contribute to the aging process are ultraviolet radiation, a diet lacking in nutritional value, and the practice of smoking. In addition to these environmental factors, genetic influences play a pivotal role in skin aging. The aging process is accompanied by a series of changes, including the degradation of subcutaneous tissue, muscles, and bones, as well as alterations in the histological and morphological structure of the skin. Notably, these changes include the atrophy of the fatty layer beneath the epidermis, a reduction in the number of fibroblasts, and a decline in the levels of intercellular matrix components, such as collagen, ceramides, hyaluronic acid, cholesterol, and sphingolipids (Table 1). These constituents are responsible for maintaining the optimal level of hydration within the epidermis, which is essential for ensuring its strength and elasticity. Consequently, the skin exhibits a dry and gray appearance. The formation of numerous wrinkles is a common phenomenon. These can be classified into two categories: deep (with a depth of greater than 0.05 mm) and superficial (up to 0.05 mm). Superficial wrinkles manifest around the eyes, whereas deep wrinkles are most prevalent in the lower regions of the face, including the cheeks and forehead. The process of cellular aging is marked by a decline in cell proliferation, leading to a reduction in the thickness of the epidermal layers [12].

### 3.1. Telomere Shortening

One of the most important theories of aging is this of telomere shortening. The unfavorable modifications that occur in telomeres depend on both extrinsic and intrinsic factors. The amount of telomere shortening has been recognized as a determinant of aging. Studies conducted on Astagalus membranaceus extract show that the active ingredients in this plant counteract such processes. According to one of the many theories on skin aging, the phenomenon of telomere shortening plays a significant role in this process. Telomeres are structural units that build chromosomes and are located at their ends. They are made up of cyclically repeated arrangements of nucleotides. They are elements that prevent chromosome damage and inappropriate genetic recombination. Telomeres shorten with each cell division, reducing the number of divisions, which leads to faster aging. The enzyme responsible for protecting against telomere shortening is telomerase. Thanks to its activity, normal tissue regeneration is possible. Telomerase deficiency leads to aging of cells and their death by apoptosis mechanism. Mutations occurring in telomeres can also be caused by the harmful effects of ultraviolet light. It contributes to increased production of reactive oxygen species, which exhibit mutagenic effects on telomeres [13,14].

### 3.2. Photoaging

Exposure to ultraviolet (UV) light has been demonstrated to increase the degradation of type I collagen, the most abundant protein in the dermis. This results in skin damage and premature aging (photoaging). UV irradiation has been shown to directly or indirectly lead to DNA damage and the formation of free radicals. These free radicals, in turn, have been observed to upregulate the expression of matrix metalloproteinases (MMPs) in skin cells. The predominant enzyme in this process is collagenase-1 (MMP-1), which is produced by a variety of cells including fibroblasts, keratinocytes, endothelial cells, macrophages, hepatocytes, chondrocytes, and osteoblasts. Following the cleavage of collagen by MMP-1, the process of degradation is further facilitated by the interstitial collagenases MMP-3 and -9. Additionally, the production of new type I procollagen (COL1) during the regeneration process is reduced by the inhibition of transforming growth factor β (TGF β)/Smad signaling and the subsequent downregulation of TGF β type II receptor (TβRII) transcription. This contributes to the wrinkled appearance of sun-exposed parts of the body, including the face and neck [15,16]. Nuclear factor kappa-β (NF-κβ), a pivotal factor in the immune–inflammatory response, has been implicated in several skin diseases, including allergic dermatitis, psoriasis vulgaris, and skin cancer. The present study hypothesizes that photoaging is significantly associated with the generation of free radicals, the stimulation of activator protein 1 and NF-κβ, and the subsequent induction of MMPs and inflammation. The transcription factor NF-κβ has been demonstrated to increase collagenase and pro-inflammatory gene expression, which in turn produces TNF-a, IL-1b, IL-6, and IL-8. Furthermore, NF-κβ has been observed to attract neutrophils containing collagenase into UVB-irradiated fibroblasts in the skin [16].

UV radiation has been classified into three distinct categories: Type A, Type B, and Type C. Type A, with a wavelength of 320–400 nm, has the greatest impact on the condition of the complexion, as it reaches the dermis. The impact of UV-A radiation on the body has been shown to include the acceleration of collagen breakdown through increased matrix metalloproteinase production, leading to tissue damage. Furthermore, UV-A has been demonstrated to exert an adverse effect on the synthesis of hyaluronic acid, which in turn gives rise to a change in the structure of proteoglycans that are found in the skin. UV-B radiation has been shown to contribute to alterations in the genetic material of keratinocytes. This form of radiation has been shown to penetrate epithelial tissue, resulting in adverse changes to the deoxyribonucleic acid. The exposure of cells to this form of radiation has been observed to result in accelerated photoaging, inflammatory reactions, destruction, and an increased susceptibility to carcinogenic effects. A hallmark of photoaging is the emergence of pigmentation spots, which are among the most frequently observed manifestations of this process. These effects are indicative of the heightened sensitivity of fibroblasts to free radicals. These changes are the result of the stimulation of the transcription of the gene responsible for melanin production. The effects of UV light on the skin are characterized by a reduction in dermal density, hyperpigmentation, formation of blackheads, development of wrinkles, loss of adequate dermal hydration, and microcirculatory disorders [13].

**Figure 2:**
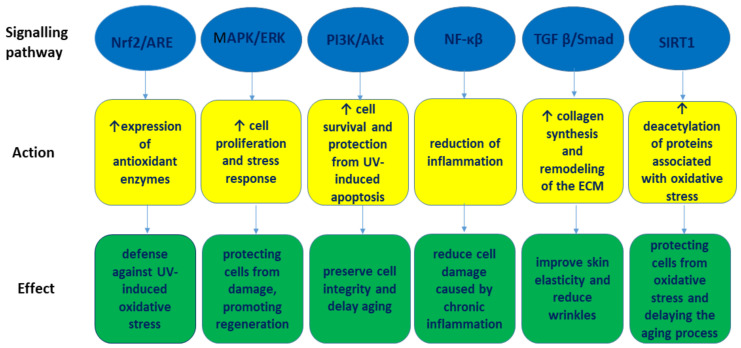
Metabolic pathways involved in the process of protection against photoaging [15,16].

The metabolic pathways activated in the photoaging protection provided by the active ingredients of Astragalus are shown in Figure 2.

### 3.3. Oxidative Stress

An increase in free radicals in vivo can result in lipid peroxidation. Malondialdehyde, the final product of lipid peroxide metabolism within cells, can serve as an indirect indicator of cellular damage. Additionally, the process of lipid peroxide formation has been observed to induce the production of antioxidant enzymes within the body. A decline in superoxide dismutase activity and antioxidant capacity within the body can lead to the attack of free radicals on normal tissues and cells, thereby accelerating the occurrence of aging and disease. Free radicals have the potential to attack normal tissues and cells, thereby accelerating the development of aging and diseases [17].

Oxidative stress has been demonstrated to play a significant role in the mechanism of aging. Free radicals have been identified as the causative agents of alterations in the extracellular matrix that occur during chronoaging, a process encompassing age-related, genetic, and hormonal aging, as well as photoaging. This impact on the formation of skin damage is a critical consideration in the broader context of aging. The intracellular metabolic processes and prolonged exposure to ultraviolet radiation have been observed to increase the production of reactive oxygen species. The presence of free radicals, including hydroxyl radicals, hydrogen peroxide, and nitric oxide, can lead to a disruption in the structure of DNA, the development of skin inflammation, a reduction in the activity of antioxidant enzymes, and the stimulation of the activator protein 1 complex. Consequently, these processes result in a reduction in collagen production and an increase in metalloproteinase activity. The net effect of these processes is the breakdown of the skin’s structural elements, which significantly accelerates the aging process. The increased production of reactive oxygen species not only results in cell damage but also in fatty acid oxidation, which accelerates the development of atherosclerosis. Consequently, this results in a reduction in the blood supply to the tissues [13,14].

### 3.4. Inflammatory Conditions

It has been demonstrated that ultraviolet radiation intensifies the production of reactive oxygen species within the body, thereby contributing to the destruction of cells, increased fatty acid oxidation, and, consequently, the initiation of tissue inflammation. Macrophages, due to their capacity for phagocytosis, are responsible for the elimination of the resulting damage. However, in instances where the inflammatory response remains uncontrolled, these cells undergo a transition in their secretion profile, releasing pro-inflammatory mediators such as interleukin 1, interleukin 6, tumor necrosis factor, acute-phase proteins, and reactive oxygen species. This heightened inflammatory response, in turn, accelerates the destruction of skin structure. As the body undergoes the natural aging process, its ability to respond to stress diminishes. Prolonged inflammation can lead to deleterious alterations in the microscopic structure of the skin tissue. A decline in the number of fibroblasts leads to a breakdown of collagen and elastin. Consequently, the epidermis undergoes a reduction in thickness and permeability, thereby impairing its capacity to safeguard against external and internal detrimental factors [13,14].

### 3.5. DNA Damage

The absorption of UV-B radiation particles results in alterations to the nucleotide sequence and the formation of DNA strands with damaged bases. This phenomenon is referred to as direct damage. In addition, exposure to UV-A radiation contributes to indirect changes. The interaction of DNA molecules with oxygen has been shown to stimulate electron and energy transfer, which in turn generates singlet oxygen ions and free radicals. Photolytic enzyme proteins are responsible for the repair of damage to the DNA structure. Skin aging is marked by a decline in the activity of genes that regulate cell proliferation, including those that are responsible for the function of fibroblasts. Consequently, the process of cell atrophy begins to take hold in the body. The rate and trajectory of the aging process are genetically predetermined. This information is stored within the cell nucleus of skin cells [13,14].

### 3.6. Impact of microRNAs

MicroRNA (miRNA) is a non-coding strand of RNA. Numerous studies have demonstrated the critical role of miRNAs transported by exosome in the process of skin renewal and rejuvenation. It has been demonstrated that these molecules facilitate the healing of damaged tissue, assist in the reduction of scarring, and promote hair growth. MiR-126 has been shown to promote wound healing by initiating a signaling cascade that stimulates cellular proliferation and metabolic activity. MiR-126 plays a pivotal role in the formation of new blood vessels and their regulation [13,14,17].

### 3.7. Accumulation of Glycation Products

The glycation process is a significant contributor to both extrinsic and intrinsic aging. Endogenous proteins present in the skin and cytoskeleton exhibit a high propensity for undergoing the glycation process. This process has been shown to contribute to the relaxation and stiffening of tissues. Notably, the glycated structural protein elastin, a hallmark of skin aging, has been observed to inappropriately bind with lysozyme, a hallmark of solar elastosis. It is noteworthy that regions not exposed to radiation are shielded from this reaction. This finding underscores the association between glycation and the photodegradation of cells. The accumulation of the end products of this mechanism leads to protein dysfunction in the skin, resulting in aging [13,14].

### 3.8. Muscle Work and Aging

The process of muscle movement contributes to the formation of facial wrinkles. The persistent contraction of these muscles results in the degradation of the structural proteins, elastin and collagen, which provide the dermis with its structural integrity. The concurrent shrinkage of fibroblasts contributes to the formation and deepening of wrinkles. The shortening of muscle fibers activates the production of contractile proteins that fix the wrinkles in place. Concurrent deterioration of the skin and subcutaneous tissues ensues. The influence of gravitational forces manifests in the form of dermal ptosis, the blurring of the jawline, the accentuation of the lines surrounding the nose and lips, the drooping of the eyelids, the sagging of the chin, the lowering of the eyebrow arches, and the appearance of wrinkles in the neck area [12].

### 3.9. Osteopenia and Osteoporosis

Bone loss is the result of an imbalance between osteoclasts and osteoblasts. As individuals age, their bones become weaker and thinner. This phenomenon is most evident in the central facial region. This decline in bone mineral density, in turn, results in a loss of its tissue support function. This phenomenon contributes to a decline in skin elasticity and the manifestation of sagging [12]. Osteopenia and osteoporosis are prevalent bone diseases that predominantly affect the elderly population. Osteoporosis, often referred to as the “silent bone thief”, is a condition marked by decreased osteoblast activity, leading to reduced bone density, weakening, and fragility. These fractures frequently result in what are termed “pathological fractures”, which, in turn, can lead to an increased disability rate among patients and even contribute to mortality. In men, osteoporosis typically manifests after the age of 70. In contrast, in women, osteoporosis is diagnosed earlier, typically after the menopause. This disparity in the onset of the disease leads to a heightened risk of complications in women [18].

### 3.10. Hormonal Changes

Hormonal regulation is a critical factor in maintaining a youthful appearance, both in females and males. In women, the male sex hormone testosterone undergoes a metabolic transformation to estrogen, while in men, testosterone is converted into dihydrotestosterone. It is important to note that the levels of these hormones decrease with age. A decline in estrogen is crucial for dermal functionality. The activation of the estrogen receptor β, which is present in fibroblasts, initiates the production of fibers, glycosaminoglycans, and intercellular matrix-filling components. Consequently, a reduction in collagen and elastin fibers occurs. The skin exhibits reduced elasticity and moisture retention, while the regenerative processes of the epidermis are impaired. Furthermore, a considerable loss of androgens occurs over time, which results in a slowing down of the activity of sebaceous secreting glands. In women undergoing menopause, a decline in estrogen has been observed to trigger alterations in the distribution of the fatty layer [12].

### 3.11. Environmental Impact

Among the various types of environmental pollutants, atmospheric smog merits particular consideration due to its composition of numerous small, fat-soluble substances. These substances have the capacity to penetrate the epidermal layer via the appendages of the skin. The interaction of these pollutants with the atmosphere has been demonstrated to promote the oxidation reaction of the skin’s components. This disruption in the renewal process of the epidermis leads to a reduction in the skin’s hydration level. Contact with air pollutants has been demonstrated to stimulate the action of pro-inflammatory factors. These substances include cytokines, leukotrienes, and prostaglandins. Interleukin 1, a member of the cytokine family, is present in epidermal cells, among others. It is imperative to acknowledge the pivotal role of IL-1 in maintaining skin homeostasis. It also plays a role in collagen and melanin production. The overproduction of prostaglandins and leukotrienes in skin tissues has the potential to enhance inflammatory reactions. Atmospheric pollutants have been demonstrated to induce an excessive production of reactive oxygen species and accelerate the process of cell death. The effects of smog on the skin include the development of acne lesions, wrinkles, accelerated aging, an increased risk of developing cancer, increased sebum secretion, hyperpigmentation, and the development of atopic dermatitis [13].

## 4. The Use of *Astragalus membranaceus* in Cosmetology and Anti-Aging Medicine

In the domain of cosmetology, Astragalus extract has been integrated into cosmetic products, endowing them with properties that promote anti-aging, photoprotective, and brightening effects. The extract has been shown to protect the skin from the development of inflammatory reactions caused by ultraviolet radiation, thereby preventing photoaging. The components of Astragalus have been shown to impede melanogenesis, a process implicated in the development of skin cancer. Consequently, the probability of sun-induced hyperpigmentation is markedly diminished [19]. A substantial body of evidence has demonstrated the epidermal hydrating and antioxidant effects of Astragalus active substances. Furthermore, studies have shown that these ingredients can improve hair condition, promote hair growth, and possess antifungal properties [20,21,22]. A study demonstrated that Astragalus cream, when used for approximately 28 days, led to an enhancement in epidermal hydration, increase in skin brightness, and reduction in the appearance of wrinkles. This determination was made through the use of corneometer measurements on the facial skin. Additionally, cutometer measurements revealed that the systematic application of the cream resulted in enhanced dermal elasticity. The study also observed that astragaloside IV, a key component of the cream, stimulated collagen synthesis, thereby contributing to the reduction of wrinkles and enhancement of skin thickness. The impact of Astragalus on dermatological disorders was substantiated in studies conducted in mice. The treatment of skin lesions with Astragalus resulted in a notable reduction in their extent. The process of exfoliation of the epidermis was found to be inhibited. Furthermore, the administration of Astragalus was found to result in a reduction in the secretion of several cytokines, including tumor necrosis factor alpha (TNF-α), IL-1, IL-6, IL-13, and other immune-specific regulators, such as P-selectin and ICAM-1. Furthermore, the administration of Astragalus extract has been demonstrated to enhance the levels of various cellular components of blood, predominantly macrophages, and to promote enhanced phagocytosis by means of upregulating lysozyme activity. The extract exhibited a soothing effect on skin inflammation by inhibiting the factor responsible for the activation of genes that induce inflammation [23,24].

In addition, the administration of Astragalus has been shown to reduce the levels of class E antibodies, which are elevated in cases of atopic dermatitis [25]. The active constituents of Astragalus, including astragaloside IV (a saponin), have been shown to impede excessive hair loss and promote hair growth. The growth cycle of hair is comprised of three phases: anagen (growth phase), catagen (transition phase), and telogen (resting phase). An experiment was conducted on rodents with regular hair loss in the resting phase, in which a single daily dose of astragaloside IV was applied to hairless skin. The results demonstrated the efficacy of Astragalus in promoting hair growth and regenerating hair follicles. Furthermore, it has been shown to delay the process of programmed epidermal cell death and to enhance the concentration of keratinocyte growth factor (KGF) [26]. Saponins, a class of compounds found in Astragalus, have been shown to promote eyebrow growth and density. In a study involving twenty women, astragaloside IV was found to stimulate the secretion of growth hormone (GH). Furthermore, the antioxidant properties of this active substance have been shown to influence hair growth phases. The antioxidant action of astragaloside IV has been shown to protect hair cells from damage induced by oxidative stress, which may contribute to a prolonged anagen phase (growth phase) of the hair. The incorporation of Astragalus extract, comprising astragaloside IV, into a cosmetic product has been shown to enhance the condition of hair follicles. This observation was further substantiated through a comparative analysis of follicle thickness between the control and experimental groups. This alteration can be attributed to the stimulation of hair follicle cell divisions and vasodilation of blood vessels, which increased the admixed delivery of nutrients [27].

Hyperscarring is defined as a skin lesion resulting from a disruption in the proliferation of fibroblasts. This results in the formation of an excess of matrix surrounding the cells, including collagen and fibronectin. This phenomenon manifests during the healing process of an epidermal break, leading to the formation of a scar that exhibits a larger surface area compared to the original wound. A range of techniques have been developed in both medical and cosmetic domains to address this condition. The treatment of scarring may be achieved through a number of methods, including surgical procedures of a relatively short duration, laser therapy, or cryotherapy. The alkaloids and phenols present in herbal extracts, including those derived from Astragalus, have been shown to modulate signaling pathways in cells, thereby impacting the development and formation of scars. For instance, a combination of Astragalus extract and red sage extract has been shown to modify the function of proteins involved in signal transduction into the cell (SMAD), thereby reducing PAI-1 gene activity [28].

Psoriasis is a chronic inflammatory condition that manifests in the form of red, scaly patches on the skin. It is characterized by three main features: acanthosis (an increase in the thickness of the horny layer of the skin), thickening of the squamous layer, and scaling of the epidermis. The active constituents of Astragalus have been shown to alleviate skin inflammation in psoriasis [6].

In addition, Astragalus has been shown to have extensive applications in the treatment of age-related diseases. The aging process is characterized by a decline in cellular and molecular integrity that accelerates with age. The goal of anti-aging medicine is to improve the physical and mental quality of life. In simplified terms, internal causes of the aging process include hormonal imbalance and inflammatory responses, while external factors include smoking, poor diet, and physical inactivity [29].

## 5. The General Mechanism of Action of *Astragalus membranaceus* in the Context of Anti-Aging

The herb Astragalus has been demonstrated to exert a beneficial influence on the majority of the mechanisms that underpin the process of aging. Telomeres are regarded as the most significant factor influencing biological aging. Astragalus contains a number of substances that stimulate telomerase and lengthen telomeres in fibroblasts [23,30,31]. These include alkaloids, saponins, polysaccharides, and cycloastragenol. The antioxidant action of Astragalus extract is a critical component of its anti-aging effect. The polysaccharides present in the extract have been shown to eliminate oxygen free radicals and augment the production of the antioxidant glutathione [20,21,26]. The antioxidant activity of Astragalus root extract is comparable to that of tocopherol-α [24]. The photoprotective effects of this herb are associated with the inhibition of metaloproteinases and the initiation of collagen I formation [21]. In addition, it has been demonstrated that Astragalus root extract inhibits the NF-κβ pathway, which is involved in matrix degradation, and reduces the UVB-induced expression of MMP-1 [20]. The skin-brightening effects of this plant are attributed to calycosin, a polysaccharide that has been demonstrated to reduce the activity of tyrosinase, melanogenesis proteins, and MITF, which are involved in melanin formation. Additionally, the process of senescence is strongly associated with the smoldering processes of chronic inflammation in tissues. Two meta-analyses have confirmed the beneficial effects of Astragalus on both humoral and cellular immunity [32] and have demonstrated a reduced secretion of pro-inflammatory mediators in patients with viral myocarditis [33]. The therapeutic effect of Astragalus (mainly cycloastragenol) in patients with psoriasis is attributable to reduced production of pro-inflammatory interleukins (IL-β1, IL-6, and IL-12). The immunomodulatory properties of this herb contribute to its antimicrobial and antitumor effects, as well as enhanced tissue regeneration. In a rodent model of inflammatory bowel disease, treatment with Astragalus resulted in a reduction in the activity of interleukin 1 beta (IL-1β) and TNFα, key inflammatory mediators, through inhibiting NF-κB activation and ERK and JNK phosphorylation. The administration of Astragalus has been demonstrated to enhance humoral immunity, as evidenced by an increase in immunoglobulin production. Furthermore, evidence suggests that Astragalus stimulates the proliferation of stem cells in the bone marrow [34,35,36].

The administration of Astragalus has been demonstrated to impede the formation of tumors through the reduction of cancer cell proliferation and the acceleration of apoptosis. Furthermore, it has been shown to inhibit the dissemination of tumor cells and to induce the accumulation of macrophages. The constituents of Astragalus have been observed to stimulate the activity of lymphocytes T and macrophages, as well as in effect the synthesis of TNF-α [36,37].

Astragalus root extract has been reported to exert a positive influence on lipid metabolism, leading to a significant reduction in total and LDL cholesterol levels. In atherosclerotic rabbits, chronic supplementation with Astragalus resulted in increased HDL cholesterol levels and a reduction in the area of aortic fatty streaks. The antiatherogenic effects of this extract have been demonstrated in apolipoprotein E-deficient mice, in which there was a significant reduction in the expression of VCAM-1, the phosphorylation of NF-κβ, and the number of macrophages in the aortic endothelium. It is noteworthy that these properties of Astragalus contribute to the enhancement of blood circulation in tissues [24,36].

In clinical settings, the administration of Astragalus extract has been demonstrated to reduce plasma glucose levels by virtue of its polysaccharide content, thereby inhibiting protein glycosylation, a process that occurs in the skin. In rat studies, Astragalus has been shown to increase protein kinase activity in the liver and reduce the amount of proteins responsible for insulin resistance. The beneficial effects of Astragalus have been primarily utilized in the adjunctive treatment of insulin resistance and type 2 diabetes. The root extract of this plant has been reported to improve diabetic kidney disease. Notably, the plant’s potential extends beyond mere skin health, as evidenced by its ability to counteract the aging process in various organs, as reported in studies by Zheng et al. [6] and Duan et al. [38].

Facial aging is a multifaceted process that encompasses a variety of physiological changes, including but not limited to the degradation of facial bones. A study demonstrated that Astragalus stimulates bone formation and prevents their breakdown. The process of osteogenesis is marked by an increase in the synthesis of bone proteins, as well as an increase in the proliferation and activity of osteoblasts. The findings of this study suggest that Astragalus may mitigate the adverse effects of postmenopausal osteoporosis, including bone fragility, chronic pain, and reduced mobility. Additionally, the plant has been observed to impede collagen degradation. Concurrently, the reduction of bone aging is associated with increased activity of the aging suppressor gene (alpha Klotho protein). The antiosteoporotic effects of Astragalus have been demonstrated in several experimental studies. In a study involving rodents with osteoporosis, the administration of Astragalus extract over a period of approximately 90 days resulted in the observed strengthening of the system of bone beams, symmetrical distribution of bone structure, and an increase in bone volume. Furthermore, an increase in mineral concentration within the bone system was observed after the oral administration of Astragalus extract at a dose of 10 µL/g [18]. Consequently, it can be utilized as an adjunct treatment in patients with osteopenia, thereby also preventing facial bone destruction [39]. Noteworthy, Astragalus exhibited beneficial effects on sustaining the effects of orthodontic treatment by increasing the rate of bone remodeling [40].

As demonstrated in the research by Awdhesh et al. [20] and Yun and Kim [21], products intended for external application that contain Astragalus have been shown to promote wound healing, epidermal regeneration, and skin vascularization.

## 6. Action of *Astragalus membranaceus* Active Substances

### 6.1. Triterpenoid Saponins

#### 6.1.1. Astragaloside IV

Astragaloside IV, a predominant saponin found in Astragalus, has garnered significant attention in research. Its anti-aging effects are associated with the activation of telomerase and its robust antioxidant properties. Research has demonstrated a correlation between the lengthening of telomeres and a retardation in facial aging. Furthermore, studies have demonstrated that astragaloside IV can prevent the degradation of fibroblasts and collagen breakdown by reducing the activity of matrix-localized metalloproteinases. The observed increase in collagen III synthesis is attributed to the interference with the functionality of Smad 7 protein, which in turn alters the activity of transforming growth factor beta (TGF-beta) [21]. Experimental studies have demonstrated that the astragaloside-related enhancement of cell and organelle autophagy plays a pivotal role in the degradation and new synthesis of collagen I [26]. Astragaloside IV has been shown to possess a variety of biological activities, including anti-inflammatory, antioxidant, antiviral, antihyperglycemic, neuroprotective, gastroprotective, antiasthmatic, and antiapoptotic properties. In a model of indomethacin-induced small intestinal inflammation, astragaloside IV has been shown to reduce the production of proinflammatory interleukins, leading to a reduction in inflammatory edema, improvement in intestinal blood circulation, and normalization of the structure of mucosa [41]. This anti-inflammatory function was hypothesized to be associated with the TLR4/NF-κβ and PERK-eIF2α pathways [24,42].

Hair follicle regression and termination of growth during catagen are accompanied by apoptosis, terminal differentiation of the proximal epithelial hair bulb, perifollicular proteolysis, and matrix remodeling. Among these phenomena, apoptosis stands as a pivotal element in the process of hair loss. Astragaloside IV has been documented to impede the Fas/Fas ligand-mediated apoptotic pathway. Furthermore, it has been observed to reduce the expression of procaspase-8, which subsequently leads to a decrease in the expression of caspase-3 and procaspase-9. Other studies have demonstrated that astragaloside IV reduces apoptosis by decreasing three mitogen-activated protein kinases (MAPKs): ERK, SAPK/JNK, and p38 phosphorylation, followed by a decrease in Bcl-2-associated X protein (Bax) expression and an increase in Bcl-2 expression. Of particular interest is the observation that the Bcl-2/Bax ratio in the hair matrix undergoes a significant decrease during catagen. Furthermore, astragaloside IV has been observed to enhance the expression of KGF, while concomitantly reducing the expression of TNF-α and IL-1β [43]. These inflammatory cytokines are associated with the terminal differentiation of keratinocytes during catagen. Consequently, astragaloside IV is regarded as a promising alternative therapeutic approach for addressing hair loss [44].

Finally, astragaloside IV inhibited the development of chronic complications of diabetes by preventing excessive accumulation of glycation end-products, e.g., pentosidine, which contributes to tissue degradation. Its concentration can be considered an indicator of the degree of development of diabetic complications [45].

#### 6.1.2. Cycloastragenol

Cycloastragenol, a triterpenoid saponin compound with steroid skeletal properties and a hydrolysis product of astragaloside IV, has been demonstrated to have multi-level effects, including anti-inflammatory, antifibrotic, and antioxidant properties. These effects include anti-inflammatory, antifibrotic, antioxidant, antibacterial, and antiviral properties. It has been shown to possess hepatoprotective, endothelioprotective, and immunoregulatory properties, which are primarily achieved through the modulation of the ERK/JNK, Wnt/ß-catenin, AKT1-mTOR-RPS6KB1, and JAK/STAT3 signaling pathways [46].

The present study examined the effects of cycloastragenol, a compound that has been demonstrated to contribute to healthy aging in female mice, on longevity. However, the available evidence does not support a role for this compound in extending mean or maximum longevity. A parallel can be drawn between the effects of cycloastragenol and astragaloside IV, as both have been observed to impede telomere shortening through the stimulation of telomerase. This effect is attributable to the regulation of the mitogen-activated protein kinase (MAPK) and protein kinase B (Akt) signaling pathways. Furthermore, this saponin has been demonstrated to diminish the DNA degradation accompanying telomere shortening. Notably, the activation of telomerase has been observed to result in enhanced glucose tolerance and reduced fasting insulin levels. These processes may be associated with the cycloastragenol-dependent ERK pathway activation in the liver. It is noteworthy that the ERK signal is attenuated by the activation of the p38 MAPK, which responds to external damage signals related to oxidative stress and aging progression. This phenomenon contributes to the development of age-related insulin resistance and glucose intolerance. Furthermore, proto-oncogene tyrosine protein kinase (c-Src), ERK kinase (MEK), and epidermal growth factor receptors have been identified as critical mediators in cycloastragenol-induced ERK phosphorylation. Consequently, cycloastragenol’s potential to activate telomerase, as well as its ability to elicit a range of cellular responses, are attributable to its modulation of the Src/MEK/ERK pathway [46,47].

The administration of cycloastragenol to mice resulted in a moderate enhancement of subcutaneous and epidermal thickness undergoing atrophy during aging. The active substance in question was observed to increase the number of erythrocytes and hemoglobin concentration in two-year-old mice. It is important to note that a decline in these red blood cell parameters is a hallmark of aging, and its potential causes include erythrocyte fragility and a deficiency in regulating stem cell pools. Noteworthy is the enhancement of telomerase, which may have beneficial effects in aged mice without an expected increase in cancer incidence. This phenomenon can be attributed to the heightened resistance of aged organisms to proliferative stimuli [48].

The anti-aging effects of cycloastragenol have been linked to an increase in the expression of the β-Klotho gene, which is induced by the activation of the ROS FGFR1-telomerase-β-Klotho signaling pathway. The Klotho gene, a subject of particular interest in this study, has been demonstrated to play a pivotal role in the regulation of telomerase activity and telomere length, thereby contributing to the retardation of the aging process. The anti-aging efficacy of cycloastragenol is further augmented by its anti-inflammatory and antioxidant properties. The anti-inflammatory effect of cycloastragenol can be attributed to its ability to enhance 5′AMP activated protein kinase (AMPK) phosphorylation. In addition, cycloastragenol has been identified as a potential target for NLRP3 inflammasomes, with the capacity to restore mitochondrial membrane loss and endoplasmic reticulum stress-induced apoptosis. This is achieved through the inhibition of caspase-1 and caspase-3 activation. Notably, cycloastragenol has been observed to elicit an immune response and impede the progression of activated immunity, a phenomenon analogous to feedback inhibition. This dual mechanism offers a potential explanation for the observed benefits of cycloastragenol in the treatment of autoimmune diseases, while concurrently hindering cancer development. The potential mechanisms underlying the observed regenerative and wound healing effects induced by cycloastragenol may be attributed to enhanced Wnt/ß-catenin expression and telomerase activation in epidermal stem cells [46].

The therapeutic effect of cycloastragenol in patients with psoriasis can be attributed to its ability to reduce the production of pro-inflammatory interleukins (IL-β1, IL-6, and IL-12). Cycloastragenol has been shown to mitigate the activity of one of the protein complexes that instigate inflammatory processes, namely the NLRP3 inflammasome. Consequently, it facilitates mitochondrial membrane reconstruction and ensures the maintenance of optimal physiological parameters in endothelial cells (e.g., normal blood pH). Furthermore, it has been demonstrated to impede the phosphorylation of interleukin alpha (IL-α). The anti-inflammatory properties of this substance have a beneficial effect on atopic skin. This is attributable to the inhibition of migration and accumulation of mast cells in specific areas of tissues, as well as the reduction in cytokine and chemokine secretion [6]. Finally, cycloastragenol, similar to the parent substance, exhibits antihyperglycemic and antiosteoporotic properties through mechanisms analogous to those previously described [47]. Furthermore, this active substance facilitates regeneration processes in patients with diabetic ulcers [2].

It is imperative to note that cycloastragenol has been demonstrated to possess no inherent toxic or genotoxic potential. A comprehensive review of five years’ worth of data, encompassing a total of 7000 person-years of use of supplements containing cycloastragenol, has yielded no reported adverse events. The supplement has been designated as Generally Recognized as Safe (GRAS) by an independent expert panel of the Food and Drug Administration [47].

### 6.2. Polysaccharides

A substantial body of evidence has demonstrated that the anti-aging effects of Astragalus polysaccharides are attributable to several mechanisms, including antioxidant, immunomodulatory, anti-inflammatory, antiatherogenic, antihyperglycemic, and telomere-protective actions. The antioxidant effects of astragalus counteract the deleterious effects of oxidative stress, which often operates discreetly in the context of metabolic processes. The overproduction of free radicals has been demonstrated to contribute to cell and tissue damage. This, in turn, accelerates the onset of aging and is an important pathogenetic factor in age-related diseases. Polysaccharides have been shown to possess antioxidant properties, including the ability to scavenge free radicals, inhibit lipid peroxidation, chelate iron ions, and enhance the activity of enzymes such as superoxide dismutase, glutathione peroxidase, and catalase. Consequently, these substances impede the production of malondialdehyde and enhance total antioxidant capacity [6].

The immunomodulatory properties of astragalus polysaccharides have been demonstrated to regulate immune function by enhancing the immune organ index (thymus and spleen), promoting the proliferation of immune cells, stimulating the release of cytokines, affecting the secretion of immunoglobulins (Ig), and modulating the conduction of immune signals. The immunomodulatory properties of Astragalus are closely associated with the reduction of inflammatory processes, which have been demonstrated to inhibit tissue aging. Polysaccharides have been shown to promote the proliferation and differentiation of B and T lymphocytes, thereby regulating the balance within the T lymphocyte subgroup. Furthermore, these polysaccharides have been shown to stimulate the growth, maturation, and function of natural killer cells and macrophages by augmenting their antigen-presenting capacity. The promotion of a humoral immune response is achieved through an increase in the synthesis of complement C3. The activation of T lymphocytes and the increased production of cytokines, including growth factors, contribute to accelerated wound healing and tissue regeneration. In experimental studies, Astragalus polysaccharides have been shown to reduce the risk of ulceration and inflammatory reactions in the oral cavity. The enhancement of tissue regeneration is achieved by stimulating the secretion of growth factors, such as fibroblast growth factor (FGF) and keratinocyte growth factor (KGF). The acceleration of wound healing by Astragalus is attributable to its capacity to stimulate epidermal regeneration, promote the fusion of damaged tissue by stimulating cell proliferation at the site of injury, and facilitate proper skin vascularization [10].

Polysaccharides have been demonstrated to enhance the production of IgA, IgG, and IgM, while concomitantly reducing the production of IgE immunoglobulins. This observation suggests the potential antiallergic properties of polysaccharides. The effects of these substances on cytokines are subject to variation depending on the status of the immune system. In physiological conditions, polysaccharides have been shown to stimulate cytokine production, primarily IL2, IL3, IL4, and IFNγ. Conversely, in instances of established inflammation accompanied by elevated cytokine levels, polysaccharides have been observed to curtail the intensity of the inflammatory response [20,21].

A dose-dependent bacteriostatic effect was exhibited by APS on the primary pathogenic bacteria, including Streptococcus, Escherichia coli, and Staphylococcus aureus [49,50]. Notably, the water-soluble fraction of the polysaccharide derived from the root of *Astragalus membranaceus* was employed in the synthesis of silver nanoparticles (AgNPs), which exhibited resistance to clinical multidrug-resistant bacteria (methicillin-resistant *Staphylococcus aureus*, methicillin-resistant *Staphylococcus epidermidis*, *Escherichia coli*, and *Pseudomonas aeruginosa*) [6,33,51]. The antiviral activity of Astragalus was confirmed against SARS-CoV-2 [10].

Moreover, the anti-aging effects of polysaccharides have been linked to the modulation of telomerase activity, the regulation of telomere-binding proteins, and the prevention of telomere shortening. Notably, these actions do not promote the development of cancer. Conversely, they have demonstrated antitumor properties by enhancing immunity, inhibiting tumor cell proliferation, inducing tumor cell apoptosis, and inhibiting tumor cell transfer. In vitro studies have demonstrated the efficacy of polysaccharides against various types of human neoplasms, including gastric, colon, lung, and breast cancer, as well as hepatoma and lymphoma [6].

Research has demonstrated that the administration of Astragalus polysaccharides has been shown to significantly extend the lifespan of the nematode *Caenorhabditis elegans* through the upregulation of microRNA (miRNA) miR-124. This, in turn, activates transcription factor 6 (ATF6) [52].

Among the various effects of polysaccharides, the induction of microRNAs (miRNAs) encoded by the miR-203a gene and the glucose-controlled protein GRP78 has been demonstrated to contribute to a decrease in insulin resistance. The antihyperglycemic effect is further enhanced by a reduction of oxidative stress in the endoplasmic reticulum. Polysaccharides have been shown to reduce protein glycosylation in hepatocytes, stimulate glucose metabolism in skeletal muscles, and increase adiponectin secretion in adipose tissue. The enhanced glucose metabolism is mediated by the activation of galectin 1 in muscle fibers. Experimental studies have demonstrated that polysaccharides offer a protective effect on pancreatic β-cells through the inhibition of cysteine protease activity, an enzyme that induces their apoptosis. In summary, the pancreatoprotective action of Astragalus polysaccharides involves a multifaceted mechanism that includes increasing tissue sensitivity to insulin, inhibiting apoptosis, and enhancing the proliferation of islet β cells, ultimately leading to a reduction in elevated blood glucose levels. The lowering of blood glucose levels contributes to anti-aging processes by inhibiting the glycation of skin matrix proteins, primarily collagen and elastin [6].

Polysaccharides have been reported to modulate the sensations of hunger and satiety in obese rats, concomitantly reducing serum leptin levels and increasing adiponectin levels [53]. Their administration has been shown to result in a significant reduction in the levels of total cholesterol, LDL cholesterol, and triglycerides in rats with non-alcoholic fatty liver disease. The findings indicate antiatherogenic effects of Astragalus polysaccharides that mitigate the age-related decrease in blood supply to tissues [54]. Notably, chronic administration of polysaccharides exhibited lipid-lowering effects comparable to those of simvastatin [6].

Metabolomic studies have demonstrated that the anti-aging effects of Astragalus polysaccharides can be attributed to amino acid metabolic pathways. The decline in the expression of genes encoding certain amino acids and their plasma levels with age has been documented in several studies [10,46]. A notable pathway involves phenylalanine, tyrosine, and tryptophan. Phenylalanine stands out as an essential amino acid, necessitating its supply through dietary means. As people age, the synthesis rate of proteins, including enzymes and hormones, slows down, along with the metabolic function of the body. Consequently, the metabolism of phenylalanine is significantly impaired [40,55]. Tyrosine, another amino acid, is derived from phenylalanine and plays a role in energy metabolism and scavenging free radicals [10]. Tyrosinase, a key enzyme in the skin’s pigmentation process, catalyzes the conversion of tyrosine to melanin, a potent antioxidant that can neutralize free radicals [6]. This amino acid can also be converted into fumaric acid and acetoacetate, thereby participating in the tricarboxylic acid cycle and providing energy for various biochemical reactions, such as oxidative stress regulation [56]. In aging rats, Astragalus polysaccharides have been shown to restore phenylalanine levels to normal, suggesting a potential role in delaying aging processes. Another salient metabolic pathway involves alanine, aspartate, and glutamate. While alanine itself lacks an antioxidant effect, it has the capacity to synthesize carnosine under in vivo conditions. Carnosine, a product of alanine, has been shown to exhibit a substantial scavenging effect on DPPH free radicals. This property of carnosine may contribute to its observed positive effect on aging skin, as reported in the study by Cho and Leung [37]. In addition, studies have found that supplementation with alanine can enhance the antioxidant capacity in mice [45]. Aspartic acid has also been demonstrated to possess antioxidant properties [52].

### 6.3. Flavonoids

#### 6.3.1. Formononetin

Flavonoids have been demonstrated to impede the cell cycle and cell growth, induce apoptosis, and exhibit a range of other biological activities, including antioxidant, osteogenic, neuroprotective, cardioprotective, antimicrobial, anticancer, antihypertensive, antiatherosclerotic, antihyperglycemic, and detoxifying effects [57,58,59].

Formononetin, an isoflavone, is classified as a phytoestrogen which has been shown to possess both estrogen-dependent and -independent mechanisms of action. The latter include antioxidant, apoptotic, anti-inflammatory, and antiproliferative effects. Preclinical and clinical studies have identified the potential of formononetin for the prevention and treatment of a number of age-related diseases, including neurodegenerative disorders (e.g., Alzheimer’s disease), obesity, and type 2 diabetes. In addition, its antitumor effects have been observed in breast, colorectal, and prostate cancers. It has been shown to impede the growth of tumors, the process of angiogenesis, and the progression of metastasis. The antidiabetic effects of formononetin are associated with an increase in tissue sensitivity to insulin. In addition, formononetin has been shown to exhibit antimicrobial properties, effectively counteracting the growth of various microorganisms, including *Mycobacterium tuberculosis*, certain members of the *Picornaviridae* family, and multiple *Candida* sp. strains [60].

In a mouse model of atopic contact dermatitis, formononetin has demonstrated significant antiallergic potential. This flavonoid has been shown to restore epithelial barrier function by inhibiting cytokines derived from epithelial cells. The observed acceleration of wound healing has been related to the angiogenic effect, which occurs through the activation of the mitogen-activated protein kinase (MAPK) and an increase in the expression of growth factors, such as the initial growth response factor 1 (Egr-1). The osteogenic action of formononetin is associated with the acceleration of osteoprogenitor cell maturation in the bone marrow [60,61].

#### 6.3.2. Calycosin

Calycosin, an isoflavone, has been found to be the most abundant in Astragalus. In recent years, calycosin has been reported to possess anticancer, antioxidative, immunomodulatory, and estrogenic-like properties [62].

Tyrosinase is an established key enzyme in melanin biosynthesis. Calycosin has been demonstrated to inhibit tyrosinase activity with an IC50 value of 38.4 µM. Given its status as a naturally occurring depigmenting agent, calycosin is a promising component for skin-brightening formulations [63].

Calycosin has been evidenced to protect against oxidative stress by inhibiting ROS generation via enhancing the activity of glutathione peroxidase, catalase, and superoxide dismutase. The antioxidant properties of this flavonoid have been attributed to the stimulation of Nrf-2 protein expression and the elevation of the levels of sirtuin 1, NOD-like receptor protein 3, and related proteins. Furthermore, calycosin has been shown to attenuate H2O2-induced apoptosis. Furthermore, calycosin has been shown to possess anti-inflammatory properties, significantly attenuating the secretion of nitric oxide, prostaglandin E2 (PGE2), TNF-α, IL-1β, and IL-6, in a mechanism that involves the NF-κβ and MAPK signal pathways’ activation. Furthermore, calycosin inhibited the development of advanced glycation end product (AGE)-induced inflammation, simultaneously reducing levels of CD68 and F4/80 mRNA, the established cell markers of inflammation [62].

Calycosin has been reported to play a role in preventing osteoporosis in postmenopausal women. The active ingredient has been shown to stimulate osteoblast differentiation by modulating the GSK-3ß pathway. Levels of specific markers of osteoblast differentiation, including alkaline phosphatase, alpha-1 type I collagen, and Runx2 protein, were significantly increased after exposure to calycosin. Furthermore, calycosin has been observed to stimulate the expression of osteoprotegerin, also known as osteoclastogenesis inhibitory factor. Correlated with the MAPK pathway, calycosin was able to abolish RANKL-induced osteoclast formation from primary bone marrow macrophages [62].

### 6.4. Other Compounds

Two isomers of hydroxyethyl derivatives of tryptophan, designated HDTIC-1 and HDTIC-2, can be isolated from *Astragalus membranaceus*. These isomers have been shown to delay the process of cellular aging through their antioxidant action, thereby preventing the excessive accumulation of glycation products and promoting cell growth and proliferation. Specifically, HDTIC has been observed to promote the transition in the cell cycle from the resting phase to the synthesis and DNA replication phase [26,64].

## 7. Effects of *Astragalus membranaceus* and Its Active Substances on Other Organs and Systems

### 7.1. Effects on the Cardiovascular System

Cycloastragenol has been demonstrated to enhance cardiac function, augment the efficiency of the heart, mitigate the consequences of cardiac ischemia, forestall cardiac tissue fibrosis, and fortify arterial vessel structure. A similar pharmacological profile is exhibited by astragaloside IV, which has been reported to restore myocardial contractility, enhance blood and oxygen supply to the heart, stimulate angiogenesis, maintain normal mitochondrial function and energy balance in cardiomyocytes. In addition, this saponin has been shown to delay the process of apoptosis, prevent cardiac fibrosis, and inhibit atherosclerotic processes in the endothelium [65,66]. Polysaccharides have been reported to regulate lipid metabolism and prevent cholesterol accumulation in blood vessels. An experimental study demonstrated that Astragalus polysaccharides enhance serum adiponectin activity and diminish leptin production, thereby reducing body weight in rats [6]. In clinical settings, the administration of Astragalus extract to patients with ischemic disease has been observed to reduce chest discomfort and enhance electrocardiographic recordings. In patients who have experienced a myocardial infarction, treatment with the extract has been shown to result in an improvement in cardiac ventricular performance. In accordance with the principles of traditional Chinese medicine, Astragalus extract is prescribed for patients with heart failure to alleviate symptoms such as dyspnea and respiratory distress [67].

### 7.2. Effects on the Nervous System

Formononetin has been demonstrated to stimulate angiogenesis in the brain. Consequently, it emerges as a promising auxiliary treatment for conditions such as Alzheimer’s disease, senile dementia, brain disorders, and depressive conditions. In animal models, formononetin has been shown to enhance memory and spatial orientation, as well as promote regeneration of dendrites and nerve fibers. Furthermore, formononetin has been shown to enhance synaptic flexibility in the brain, a process that plays a pivotal role in learning, memory, and adaptation to novel conditions. A comparable mechanism has been reported for cycloastragenol, which has been shown to impede the aggregation of amyloid proteins. Additionally, this saponin has demonstrated antidepressant properties and mitigated the severity of stroke-related damage. Astragaloside IV has demonstrated neuroprotective properties, thereby enhancing memory and cognitive abilities [68].

### 7.3. Effects on the Respiratory System

The utilization of Astragalus extract in an add-on therapeutic modality has been demonstrated to result in the reinforcement of the alveolar structure, the diminution of the area affected by pulmonary fibrosis, and the reduction of the risk of pulmonary edema development. The anti-inflammatory, antiallergic, and immunomodulatory properties of Astragalus have been shown to reduce the risk of respiratory infections and asthma attacks. In human subjects, a daily supplementation regimen of 8 g of Astragalus extract over a period of approximately 60 days has been shown to enhance interferon synthesis. In murine models, the administration of Astragalus was observed to reduce bronchial inflammation, as well as to prevent bronchospasm and bronchial remodeling [69,70,71].

### 7.4. Effects on the Liver and Kidneys

Cycloastragenol has been identified as an activator of the farnesoid X receptor (FXR). This receptor has been demonstrated to play a pivotal role in enhancing fat metabolism and sustaining normal plasma glucose and cholesterol levels. In the liver, the activation of this receptor has been demonstrated to prevent the development of inflammation, steatosis, and fibrosis. In hepatocytes, cycloastragenol has been shown to reduce glucose and fat content, as well as to decrease excessive bile acid production [46]. Moreover, polysaccharides have been shown to regulate the concentrations of alanine aminotransferase, aspartate aminotransferase, and alkaline phosphatase [9]. In a separate study, rats with kidney injury were observed to demonstrate alleviation of inflammatory processes, reduction of plasma urea concentrations, stimulation of nephron regeneration, and facilitation of the formation of new renal tubules following treatment with Astragalus [9,53].

### 7.5. Effects on Physical Activity

The administration of Astragalus (*Astragalus membranaceus*) root extract has been demonstrated to enhance athletic performance and physical endurance. Moreover, the root has been reported to expedite the process of tissue regeneration and mitigate the adverse effects of physical exertion on immune system function and cortisol elevation. The active substances in Astragalus have been shown to dilate blood vessels, thereby increasing the oxygen supply to tissues. Furthermore, they have been demonstrated to reduce the formation of oxygen free radicals, increase the production of erythrocytes, and accelerate the body’s adaptation to new conditions. The ability of Astragalus to enhance the elimination of metabolites, including lactic acid, has been shown to delay the onset of fatigue in muscle cells. It is widely recommended as a dietary supplement for individuals engaged in endurance sports. However, the efficacy of Astragalus in promoting muscle tissue expansion remains inconclusive [72].

### 7.6. Effects on Menopause and Fertility

In women experiencing symptoms related to the menopausal transition, the administration of Astragalus extract over a period of approximately three months has been shown to stimulate estrogen secretion and to prevent the onset of osteoporosis by increasing the proliferation of osteoblasts. In younger women, the administration of Astragalus supplementation has been associated with increased plasma estrogen levels, elevated ovulation rates, and augmented endometrial thickness. Concurrently, it was observed that the probability of successful implantation of the embryo and the subsequent maintenance of the pregnancy was enhanced [73,74]. Astragalus has also shown beneficial effects in male infertility, increasing sperm count and improving sperm parameters [75].

The following schematic overview provides a structured and detailed insight into how *Astragalus membranaceus* exerts protective effects against cellular aging processes (Table 2).

The diverse mechanisms of *Astragalus membranaceus* contribute to its potential anti-aging properties, targeting telomere maintenance, oxidative stress reduction, immune modulation, and metabolic regulation. Future clinical trials are necessary to further validate its efficacy in human aging and longevity.

## 8. Adverse Effects and Safety Considerations of *Astragalus membranaceus*

*Astragalus membranaceus* is widely recognized for its immunomodulatory, antioxidant, and anti-aging properties, with a strong history of use in traditional medicine. While generally well tolerated, certain adverse effects and contraindications have been reported, necessitating careful consideration, particularly in specific populations or when used concomitantly with other medications.

Among the most frequently reported side effects are mild allergic reactions, including skin rashes, itching, and nasal congestion. Some individuals have also experienced gastrointestinal discomfort, such as nausea, bloating, and diarrhea, particularly when consuming high doses of Astragalus extracts. Although these effects are generally mild and self-limiting, they may discourage continued use in sensitive individuals [76,77].

One of the primary concerns associated with Astragalus supplementation is its potential interaction with certain medications. Due to its immunostimulatory properties, Astragalus may reduce the efficacy of immunosuppressive drugs, such as cyclosporine and tacrolimus, posing a risk for individuals who have undergone organ transplantation or are receiving treatment for autoimmune diseases [78]. Furthermore, its ability to enhance blood circulation and fibrinolysis raises concerns regarding an increased risk of bleeding when used alongside anticoagulant or antiplatelet drugs, including warfarin, aspirin, and clopidogrel [79]. Additionally, Astragalus-derived polysaccharides have demonstrated hypoglycemic effects, which, while beneficial for metabolic health, may potentiate the action of antidiabetic medications, increasing the risk of hypoglycemia in susceptible individuals [80].

Beyond pharmacological interactions, certain environmental and metabolic factors influence the safety profile of Astragalus. In regions where soil is rich in selenium, Astragalus species may accumulate toxic levels of this trace element. Excessive selenium intake has been linked to neurological dysfunction, including muscle weakness and paralysis, highlighting the importance of source control in dietary and therapeutic applications [81]. Certain populations require special precautions when considering Astragalus supplementation. Due to insufficient clinical data regarding its safety during pregnancy and lactation, its use is not recommended for pregnant or breastfeeding women [82]. Similarly, individuals with autoimmune diseases, such as lupus, multiple sclerosis, or rheumatoid arthritis, may experience exacerbation of symptoms due to the plant’s immune-activating effects [77]. Patients undergoing post-organ transplantation should also avoid Astragalus, as its immunostimulatory properties could interfere with the necessary suppression of immune responses, increasing the likelihood of organ rejection [78].

Despite these considerations, *Astragalus membranaceus* remains a widely used and well-tolerated medicinal herb, with the majority of users experiencing no significant adverse effects. Nevertheless, individuals with pre-existing health conditions or those taking concurrent medications should seek professional medical advice before incorporating Astragalus into their regimen. A thorough understanding of its pharmacological interactions, contraindications, and potential toxicities is essential for optimizing its therapeutic benefits while minimizing risks.

## 9. Translational Relevance of *Astragalus membranaceus* in Cellular Senescence

A significant proportion of studies investigating the effects of *Astragalus membranaceus* on cellular senescence have been conducted in vitro or in animal models. While these studies provide essential mechanistic insights, their direct applicability to human aging remains a subject of ongoing investigation. The biological processes involved in senescence, such as oxidative stress, inflammation, and telomere attrition, are conserved across species; however, differences in metabolism, pharmacokinetics, and genetic variability may influence the clinical efficacy of Astragalus extracts in humans [76,80].

Preclinical studies have demonstrated that active compounds such as astragaloside IV, cycloastragenol, and flavonoids exert antioxidant, anti-inflammatory, and telomere-protective effects (Table 1). Astragaloside IV has been shown to enhance telomerase activity in vitro, suggesting a potential role in telomere length maintenance [82]. However, the doses used in these experiments often exceed those achievable through oral supplementation in humans. In animal models, cycloastragenol has been reported to reduce markers of senescence-associated secretory phenotype (SASP) and enhance mitochondrial function, yet whether these effects translate into measurable improvements in human aging remains unclear [81].

### 9.1. Variations in Study Results and Inconsistencies

The efficacy of *Astragalus membranaceus* and its active compounds varies across studies due to differences in the following:

1. Extract composition and standardization—Many studies use different extraction methods, leading to variability in the concentration of bioactive compounds. For instance, some studies focus on polysaccharides, while others emphasize saponins like astragaloside IV or flavonoids such as calycosin [78].
2. Study models and experimental conditions—While some in vitro studies report significant telomerase activation, others show only moderate effects depending on the type of cell line used. Similarly, in vivo studies demonstrate age-related benefits primarily in metabolic and immune function rather than direct telomere elongation [77].
3. Interindividual variability—Human studies report differing responses based on genetic and lifestyle factors. Some trials show enhanced immune function, while others find minimal effects on inflammatory markers [79].

### 9.2. Limitations of Preclinical Models and Clinical Evidence

Rodent models provide valuable insights into systemic effects, but their relatively short lifespan and distinct metabolic pathways may not fully replicate the complex aging processes observed in humans. Moreover, differences in drug metabolism between rodents and humans can result in discrepancies in efficacy and toxicity profiles [78]. While Astragalus has demonstrated beneficial effects in modulating inflammatory and oxidative stress pathways in aged mice, clinical studies evaluating its long-term impact on aging biomarkers in human subjects are sparse.

To date, clinical studies on *Astragalus membranaceus* have primarily focused on immune function, glucose metabolism, and skin regeneration. The observed increase in interferon synthesis and improved markers of insulin sensitivity suggest systemic benefits beyond localized cellular effects [77]. The reported enhancement of fibroblast activity and collagen deposition in dermatological applications further supports the regenerative potential of Astragalus-derived compounds. However, variations in formulation bioavailability, penetration depth, and individual skin responses necessitate further controlled trials to confirm efficacy and optimize delivery methods.

### 9.3. Knowledge Gaps and Future Directions

Despite promising results, several gaps remain in our understanding of *Astragalus membranaceus* in human aging:

1. Longitudinal studies on aging biomarkers—Most human studies focus on short-term effects. There is a need for long-term trials assessing parameters such as telomere dynamics, DNA methylation age, and senescence-associated secretory phenotype (SASP).
2. Mechanistic understanding in humans—While in vitro and animal models suggest that astragaloside IV and cycloastragenol modulate telomerase, the extent of this effect in human subjects remains uncertain [82].
3. Optimized dosage and bioavailability—Astragaloside IV and cycloastragenol exhibit low oral bioavailability. Future research should explore advanced delivery systems, such as liposomal formulations or nanoparticles, to enhance absorption and efficacy [79].
4. Comparative effectiveness studies—More studies are needed comparing Astragalus supplementation with other anti-aging interventions, such as senolytics or caloric restriction mimetics, to determine its relative efficacy in delaying senescence.

### 9.4. Bridging the Gap Between Preclinical and Clinical Research

To enhance translational relevance, future research should address the following key aspects:

1. Standardized extracts and dosing: Variability in Astragalus extract composition affects bioavailability and efficacy. Studies using standardized formulations with defined astragaloside IV and cycloastragenol concentrations are essential.
2. Validated aging biomarkers: Current research relies on indirect markers such as oxidative stress and inflammation. Clinical trials should incorporate validated aging biomarkers, such as telomere length, DNA methylation age, and SASP components, to assess the true impact of Astragalus on aging.
3. Longitudinal human studies: Most clinical data are derived from short-term interventions. Long-term studies evaluating functional aging parameters (e.g., cognitive function, skin elasticity, and metabolic health) are needed. A brief description of currently available clinical trials is included in Table 3.
4. Interindividual variability: Genetic and epigenetic differences may influence individual responses to Astragalus. Future studies should explore the role of genetic polymorphisms in modulating its effects on aging pathways.

By addressing these challenges, *Astragalus membranaceus* could transition from a promising nutraceutical to a clinically validated intervention for age-related conditions.

## 10. Conclusions

*Astragalus membranaceus* has been suggested to have potential applications in both cosmetology and anti-aging medicine. Its active constituents may contribute to diverse health benefits, potentially affecting multiple organs and systems. However, it is important to acknowledge that other botanicals containing saponins, polysaccharides, or flavonoids might exert similar effects. A distinguishing feature of Astragalus is the proposed mechanism of action of certain saponins (cycloastragenol and astragaloside IV), which have been suggested to play a role in mitigating age-related telomere shortening. The herb’s multifaceted anti-aging profile appears to include antioxidant, immunomodulatory, and anti-inflammatory properties. Given that cancers are among the most significant factors influencing life expectancy, the immunomodulatory and potentially anticancer effects of Astragalus have been hypothesized to contribute to longevity. Nevertheless, it is crucial to emphasize that the concept of anti-aging extends beyond lifespan extension to encompass the maintenance of physical, mental, and cognitive function. The potential effects of Astragalus align with the broader definition of prolonging health span.

The proposed protective role of Astragalus against external factors, such as environmental stress and ultraviolet radiation, along with its anti-inflammatory, regenerative, skin-brightening, and antibacterial properties, may have a positive impact on skin condition. The herb is frequently incorporated into anti-acne and brightening creams, face gels, and lotions. These formulations are used across a range of skin types, including dry and oily skin, and by individuals across different age groups.

However, it is essential to highlight that most research on *Astragalus membranaceus* has been conducted in in vitro and in vivo experimental models. While a limited number of clinical trials have been performed (summarized in Table 3), the available evidence remains insufficient to draw definitive conclusions regarding its clinical efficacy. There is a clear need for well-designed, randomized clinical trials to validate the findings of preclinical studies and to assess their translational relevance in human populations.

Additionally, current data do not indicate significant adverse effects following prolonged supplementation with Astragalus and its active constituents. However, the lack of large-scale randomized clinical trials and meta-analyses systematically evaluating the herb’s effects on cellular metabolism, aging processes, and age-related conditions represents a critical limitation. Until further evidence is available, conclusions regarding its clinical benefits should be drawn with caution.

## Figures and Tables

**Table 1:** Cellular changes in mature skin [12].

Cellular Change	Description	Implication for Skin Aging
Decreased number of keratinocytes	Keratinocytes, responsible for the formation of the epidermis, decrease in number with age.	Leads to thinning of the epidermis, reduced barrier function, and increased permeability, contributing to skin dryness and sensitivity
Decreased number and functionality of fibroblasts	Fibroblasts are essential for collagen production; their number and activity decrease with age.	Results in the loss of dermal structure, decreased skin elasticity, and formation of wrinkles.
Impaired synthesis of type I and type III collagen	Collagen is a major structural protein in the dermis. Aging leads to reduced synthesis of type I and III collagen.	Leads to thinning of the skin, formation of fine lines, and loss of firmness.
Impaired synthesis of elastin	Elastin, which provides skin elasticity, is also produced in lesser quantities as we age.	Skin becomes less elastic, resulting in sagging and the formation of wrinkles.
Decreased number of melanocytes	Melanocytes, responsible for pigment production, decrease in number with age.	Can lead to uneven pigmentation, the appearance of age spots, and reduced skin tone.
Decreased number and functionality of Langerhans cells	Langerhans cells, part of the skin’s immune system, decrease with age, reducing the skin’s ability to mount immune responses.	Results in a decreased ability to protect the skin from pathogens, increasing susceptibility to infections and inflammatory responses.
Decreased number and functionality of dendritic cells	Dendritic cells play a crucial role in immune surveillance. Their decline contributes to impaired immune responses.	Increases susceptibility to infections and skin disorders, while reducing the efficiency of the skin’s immune defense mechanisms.
Decreased number of mast cells	Mast cells, involved in immune responses and inflammation, decrease in number and function with age.	Impaired inflammatory responses and reduced capacity to repair skin damage.
Decreased toll-like receptor (TLR) activity (innate immunity)	Toll-like receptors play a key role in the innate immune response. Their decreased activity affects the skin’s ability to detect pathogens.	Impaired detection of pathogens and a decreased inflammatory response to skin damage.
Decreased secretion of antimicrobial proteins	Skin produces antimicrobial proteins to protect against infections. Their secretion declines with age.	Results in the loss of dermal structure, decreased skin elasticity, and formation of wrinkles.

**Table 2:** Schematic representation of *Astragalus membranaceus* mechanisms in cellular aging.

Key Bioactive Compounds and Their Effects
Astragaloside IV and cycloastragenol Activate telomerase (TERT), extending telomere length and delaying cellular senescence. Enhance mitochondrial function, reducing ROS levels and oxidative stress. Calycosin and formononetin Modulate NF-κB signaling, decreasing pro-inflammatory cytokine production (IL-6, TNF-α, and IL-1β). Stimulate SIRT1/AMPK pathway, improving metabolic efficiency and cellular longevity. Astragalus polysaccharides Enhance immune function, increasing T-cell activity and phagocytosis. Reduce protein glycosylation, mitigating age-related metabolic dysfunctions.
**Cellular pathways targeted by *Astragalus membranaceus***
Telomere protection and DNA stability ○ Cycloastragenol upregulates TERT, delaying replicative senescence. ○ Reduction of DNA damage markers (γ-H2AX and p53 activation). Oxidative stress and mitochondrial homeostasis ○ Activation of Nrf2 pathway, promoting antioxidant enzyme production (e.g., SOD and catalase). ○ Enhancement of mitochondrial biogenesis and function, reducing ROS-induced cellular damage. Inflammation and immune regulation ○ Suppression of NF-κB signaling, leading to decreased inflammatory responses. ○ Increased secretion of anti-inflammatory cytokines (IL-10 and TGF-β). Autophagy and apoptosis modulation ○ Stimulation of autophagic clearance (LC3-II upregulation), reducing cellular debris accumulation. ○ Regulation of Bcl-2/Bax ratio, preventing excessive apoptosis and maintaining cell viability. Metabolic and endocrine influence ○ Upregulation of AMPK/SIRT1/mTOR pathways, enhancing metabolic balance and energy production. ○ Reduction of insulin resistance and glycation end-products, beneficial for longevity and skin aging.

**Table 3:** Research on the anti-aging effects of *Astragalus membranaceus* in a clinical setting.

Subject of Study	Type of Study	Treatment	Outcome	Reference
Astragaloside IV and GH secretion	Clinical study (20 women)	Astragaloside IV supplementation	Increased growth hormone secretion, prolonged anagen phase of hair growth	[27]
Astragalus and immunity	Meta-analysis	Astragalus extract	Improved humoral and cellular immunity	[32]
Astragalus and viral myocarditis	Meta-analysis	Astragalus extract	Reduced secretion of pro-inflammatory mediators	[33]
Astragalus and glucose metabolism	Clinical study	Astragalus extract	Lowered plasma glucose levels, inhibition of protein glycosylation	[6]
Astragalus and bone remodeling	Clinical study	Astragalus extract	Increased rate of bone remodeling, sustained orthodontic treatment effects	[40]
Cycloastragenol and psoriasis	Clinical study	Cycloastragenol	Reduction of pro-inflammatory interleukins (IL-β1, IL-6, IL-12)	[6]
Astragalus and diabetic ulcers	Clinical study	Astragalus extract	Enhanced regeneration in diabetic ulcers	[2]
Formononetin and aging diseases	Preclinical and clinical studies	Formononetin	Prevention of neurodegenerative disorders, obesity, type 2 diabetes	[60]
Calycosin and osteoporosis	Clinical study	Calycosin	Stimulated osteoblast differentiation, increased markers of osteoblast differentiation	[62]
Astragalus and interferon synthesis	Clinical study	8 g Astragalus extract daily for ~60 days	Enhanced interferon synthesis	[71]
Astragalus and menopause	Clinical study	Astragalus extract for ~3 months	Stimulated estrogen secretion, increased osteoblast proliferation	[74]
Astragalus and male infertility	Clinical study	Astragalus supplementation	Increased sperm count, improved sperm parameters	[75]

## Data Availability

The data presented in this study are available on request from the corresponding author.
